# Professional competence required in advanced practice nursing in critical care: An exploratory qualitative study

**DOI:** 10.1002/nop2.2032

**Published:** 2023-10-11

**Authors:** Marianne Trygg Solberg, Ingunn Pedersen, Cathrine Mathisen, Inger Johanne Finnstrøm, Per Kristian Lundin, Andréa Aparecida Gonçalves Nes

**Affiliations:** ^1^ Lovisenberg Diaconal University College Department for Postgraduate Studies Oslo Norway; ^2^ University of North Bodø Norway; ^3^ Bærum hospital, Intensive Care Unit, Vestre Viken HF Bærum Norway; ^4^ Intensive Care, Section 1, Rikshospitalet Department of Postoperative and Intensive Care Division of Emergencies and Critical Care Oslo University Hospital Oslo Norway; ^5^ Department for Bachelor Education Lovisenberg Diaconal University College Oslo Norway

**Keywords:** advanced practice nursing, critical care, master nursing education, professional competence, qualitative study

## Abstract

**Aim:**

To identify the required competencies of advanced practice nurses (APNs) working with patients in critical care units in Norway.

**Design:**

An exploratory qualitative design.

**Methods:**

Four focus group interviews were performed with 18 nurses who worked in critical care units. The data were examined by inductive content analysis following Graneheim and Lundman's approach.

**Findings:**

Our study found that APNs in critical care require the following professional competencies to meet the needs of patients characterised by greater age, comorbidities and increased complexity: (1) intrapersonal skills as revealed in the subthemes of self‐awareness; motivation and commitment; strong mental health and upholding ethical standards, (2) advanced clinical decision‐making skills as identified in the subthemes of integration of theory and practice; complex practical and technical skills; dealing with increased delegated responsibility and taking the lead in managing increased practice complexity and (3) interpersonal skills, including peer guidance, practising collaboratively and the ability to position oneself.

## 1 INTRODUCTION

Health care is rapidly changing globally and has become increasingly specialised and complex (International Council of Nurses, 2020; Øvrebø et al., 2022). Critical care nurses (CCNs) provide care to acutely or critically ill patients who are characterised by an increased degree of complexity and comorbidity (Egerod et al., 2021; Woo et al., 2017). CCNs' professional competence is crucial to ensuring high‐quality nursing in critical care, as they must work independently, safely and effectively in constantly changing situations (Ääri et al., 2008; Egerod et al., 2021; Waters et al., 2013). It has been suggested that to achieve the necessary competence, CCNs should receive additional education in critical care in their master's degree programme to qualify as advanced practice nurses (APNs) in critical care (Woo et al., 2017). The role of an APN, whose credentials include 120 credits in the European Credit Transfer System (Egerod et al., 2021), incorporates expert knowledge and skills to perform complex decision‐making in advanced nursing in the clinical context (International Council of Nurses, 2020; Tracy et al., 2023). Credential levels and core competencies vary according to workplace and education degree (Egerod et al., 2021; International Council of Nurses, 2020), see some examples in Table 1.

**TABLE 1:** Overview of nursing credential levels and professional core competencies.

Terminology	Education degree	Gradually development of core competencies
Registered nurse (RN)	Bachelor, 180 European Credit Transfer System	Principles of nursing care Clinical guidelines Nursing interventions (Ääri et al., 2008)
Critical care nurse (CNN)	RN with education in intensive and critical care, 90 European Credit Transfer System	Ethical activity (sensitiveness) Decision making Development work (Evidence‐based practice) Collaboration (Ääri et al., 2008)
Advanced practice nurse (APN)	RN with a master's degree, 120 European Credit Transfer System	Ethical decision‐making skills Guidance and coaching Evidence‐based practice Leadership Consultation (Tracy et al., 2023)

In a Norwegian master's programme in APN, it is necessary to develop learning outcomes aligned with contemporary standards for critically ill patient care, to provide APNs with the needed professional competence. The term *professional competence*, including professional competence in CCNs, describes a phenomenon that is variously and inconsistently defined (Glaesser, 2019). Professional competence in CCNs is perceived as multidimensional (Lakanmaa et al., 2012) and difficult to describe, as CCNs manage many distinct tasks within a diverse spectrum of contexts in clinical practice (Ääri et al., 2008; Dunn et al., 2000). Nevertheless, the concept of professional competence in CCNs embraces teamwork, decision‐making and being able to manage situations in addition to empathising with patients beyond the technical aspects of care (DeGrande et al., 2018). It is also essential that CCNs have core qualities such as personal maturity and a good attitude (Henriksen et al., 2021; Lindberg, 2006). In contrast to the professional competencies of CCNs, there are no appropriate descriptions of the competencies required of APNs in critical care in Europe (Egerod et al., 2021).

Regarding education, the European Union Qualifications Framework (Waters et al., 2013) prescribes that the education of CCNs take a competence‐based approach and the European Federation of CCN Associations (EfCCNa) has published a framework‐of‐competency tool for educators' use that describes key requirements in four domains: (1) clinical, (2) professional, (3) managerial and (4) educational and developmental (Waters et al., 2013). A master's programme for APNs in critical care is intended to go further, however, to cultivate nurses who are able to integrate advanced theoretical knowledge with practical and interpersonal skills in caring for vulnerable, critically ill patients (DeGrande et al., 2018; Øvrebø et al., 2022). To do so, such programmes must align with the scope of practice and competencies expected of APNs (International Council of Nurses, 2020). Nevertheless, the required competencies for APNs in the critical care context are not specified in the EfCCNa document, and, to the best of our knowledge, no studies have investigated this theme. Thus, this study explored the required competencies of APNs working with patients in critical care units in Norway.

## 2 METHODS

Investigating a profession's competencies requires discovering the workers' understanding of their work (Lindberg, 2006). Therefore, this study adopted an exploratory qualitative design with focus group interview, which is often used to identify the perspectives of experienced professionals (Polit & Beck, 2020). The group dynamic in focus group interviews might stimulate spontaneous expressive and emotional views that can provide rich information in an efficient manner (Polit & Beck, 2020). This study follows the consolidated criteria for reporting qualitative research (COREQ guidelines; Tong et al., 2007).

### 2.1 Setting and participants

The interviews were conducted in four critical care units, three in university hospitals and one at a regional hospital in or near Oslo, Norway. These units were chosen as they offer a clinical practice with advanced intensive care for patients with increased complexity and comorbidity. The first author contacted the charge nurse in the chosen units to support the recruiting process. Those invited all nurses employed to share their experiences in focus group interviews. To participate in the study, they need to agree to and sign the study's written information.

### 2.2 Ethical approval and informed consent

Permission to conduct the study was obtained from the Norwegian Centre for Research Data (57891). The participants were given written and verbal information about the aim of the study. Participation was voluntary and adhered to the Declaration of Helsinki. All the participants provided written informed consent after being told that they could withdraw at any time before the analysis.

### 2.3 Data collection

The interviews were conducted according to expert recommendations (Polit & Beck, 2020) in February–May 2018. Each interview lasted around 60 min. The first author moderated all the interviews with the assistance of two of the co‐authors as co‐moderators (IJF and CM). The moderator welcomed the participants, restated the aim of the study and emphasised that there were no right or wrong answers to the questions. We used a semi‐structured interview guide within all the groups, and the main question concerned the participants' clinical practice experiences and addressed which competencies they believed an APN needed in day‐to‐day work. Prepared follow‐up questions covered topics such as knowledge, skills, general competencies and whether they had experienced different competency needs in recent years than previously. The interview guide was carefully developed by experienced researchers, but not piloted. The co‐moderators took notes and asked clarifying questions at the end of the interview. The interviews were audiotaped and subsequently transcribed verbatim by the first and third authors.

### 2.4 Data analysis

We adopted Graneheim and Lundman (2004) inductive content approach to analyse the data in three steps: (1) the transcripts were read several times to gain insight into the content; (2) the text was condensed into meaning units with descriptions close to the text; (3) the codes were inductively developed by reading and rereading the meaning units. After several discussion meetings, a consensus was reached on categorising the findings into categories and sub‐categories (Table 2). All the authors participated in the whole analysis process.

**TABLE 2:** Examples of the analysis process from meaning unit to theme.

Raw data is divided into meaning units	Condensed meaning unit description close to the text	Interpretation of the underlying meaning	Sub‐categories	Category
I also think the responsibility is probably more today than before, so I think as long as you like it and think it's challenging and feel safe, then it's perfectly fine, but I think the responsibility is probably much more on us now than it was before.	The responsibility is much more on us now than before. If you like it, find it challenging and feel safe, it's fine.	Increased responsibility is delegated to intensive care nurses.	Dealing with increased delegated responsibility	Advanced clinical decision‐making skills
These tough personal things that you just have to go straight into, i.e. people who are having a tough time or people who get married 2–3 h before they die and other quite emotionally strong things, I must say, sometimes I think, ‘Am I at the theatre?’ It's unbelievable that it can be like this; you cannot explain to anyone outside the hospital what it's like, because it's completely impossible to imagine. It's just completely absurd; sometimes, I get a bit put out. [Several agreed.]	There are many different tough personal things that you simply must go straight into. In the midst of it, you get the feeling of being in a theatre. You cannot explain to anyone outside the intensive care unit what it's like, because it's completely impossible to grasp.	APNs must have the skills to deal with situations that are far from experiences in their own lives. They must cope with unimaginable situations that can cause psychological stress and must have the ability to process unimaginable events. It requires guidance and cognitive work not to take destructive thoughts with you further. Be mentally strong.	Strong mental health	Intrapersonal skills
Regarding the ventilator treatment: So, I think that maybe we have not been left behind, but we have been given a greater opportunity to become better at gaining more knowledge, and perhaps have an even closer dialogue with the doctor in charge, At least I can feel that there is a development that has happened in me, but I have a few years' experience. (One participant agreed verbally)	I have long experience, and I acknowledge a development in me, that we have a greater opportunity, compared to earlier, to gain more knowledge and become smarter and achieve a closer dialogue with the doctor in charge.	The critical care nurse's responsibility regarding ventilator treatment has changed, giving them more knowledge to influence decisions about ventilator treatment.	Ability to position oneself	Interpersonal skills

### 2.5 Trustworthiness

Following Polit and Beck (2020), the data collection was conducted in a conference room familiar to the participants at each hospital. The moderator avoided reacting to the participants and tried not to influence their answers. The participants talked at length about their experiences and were not afraid to express their diverse perceptions. Evaluation of data saturation was based on the research perception that no new information was obtained in the fourth interview, and redundancy of the collected data arose. The co‐moderators used their notes from the interviews to supplement the verbal transcript in the analysis discussions. All the authors except the last author are APNs in critical care. The first, second and third authors work in a postgraduate critical care nursing education programme, the fourth and fifth work at a general critical care unit and the last author has research and clinical experience with patients in critical and chronic condition. The authors' broad, diverse expertise (extensive research and clinical experience) deepened the trustworthiness of the interpretation, as all the authors participated in the analysis and agreed on the findings. The researchers reflexibility was maintained throughout the analysis‐ and the article writing process by asking about their thoughts and feelings about the data, the codes and the final findings. The first, second and last authors have collaborated conscientiously to include all the comments and suggestions from the author team, especially from the third and fourth authors who still work as APN in critical care.

## 3 FINDINGS

Eighteen nurses agreed to participate (17 females; 1 male). The four focus groups comprised four, four, six and four participants. Table 3 shows the participants' expertise and years of experience (range: 1–34) in critical care units.

**TABLE 3:** The participants' expertise.

Participant number	Specialist competencies	Years of experience in critical care units
1	Anaesthesia	5
2	Intensive and critical care	10
3	Intensive and critical care	27
4	Anaesthesia	10
5	APN^a^ in critical care	7.5
6	Intensive and critical care	15
7	Intensive and critical care	16
8	APN in critical care	8
9	Intensive and critical care	31
10	Intensive and critical care	9
11	APN in critical care	1
12	APN in critical care	Unknown
13	Intensive and critical care	34
14	Intensive and critical care	Unknown
15	Intensive and critical care	12
16	Intensive and critical care	29
17	Intensive and critical care	7
18	Intensive and critical care	8

^a^ Advanced practice nurse.

The data yielded three overall themes reflecting the required competencies of APNs in critical care as experienced by the participants (Table 4). The data show that the participants were concerned about years of experience, professional environment, departmental culture, leaders' facilitation and level of peers' education, as they perceived that these factors influenced their professional competence.

**TABLE 4:** Category and sub‐categories reflecting the APNs' needed professional competence.

Intrapersonal skills	Advanced clinical decision‐making skills	Interpersonal skills
Self‐awareness	Integration of theory and practice	Practicing collaboratively
Motivation and commitment	Complex practical and technical skills	Ability to position oneself
Strong mental health	Dealing with increased delegated responsibility	Peer guidance
Upholding of ethical standards	Taking the lead in managing increased practice complexity	

The findings are presented according to the main categories of skills and knowledge (Table 4). To ensure anonymity in references to individual participants' statements, non‐identifying numbers are used.

### 3.1 Intrapersonal skills

A prominent finding under the category of intrapersonal skills was that APNs should possess *self‐awareness* regarding their professional limitations. APNs must take responsibility for their actions and be open, interested, interrogative and willing to ask for help when needed. The participants indicated that excessive self‐confidence may negatively affect an APN's clinical practice. APNs must maintain up‐to‐date professional knowledge and skills that are visible to physicians and colleagues, which requires hard work. Several participants mentioned weighty requirements in practice that they must consider and prioritise. As one participant stated, ‘You have to be humble and reflect on why you do as you do, not just blindly follow a [physician's] regulation. You must reflect and may be accompanied by someone regarding almost everything you do. You are terrified to do something wrong, so you start thinking about why you are doing it and what the consequences may be’ (11). Regarding self‐awareness, APNs must also be aware of their own reactions in diverse situations and must control their feelings if they become annoyed. Instead of being defensive, they must be open to criticism and questions from colleagues and patients' relatives. The participants believed that guidance was an important means of gaining self‐awareness and recognising their own limitations.

There was strong agreement that APNs must be *motivated* to quickly acquire new theoretical knowledge, skills and techniques and to master the many types of technical equipment they handle. An experienced participant explained, ‘You must have an interest and a desire to be a good APN; your commitment is very important. You must try to be as good as you can, not relax because you have done what you are supposed to. You should think about how you can get a little further today; for example, can I manage to step down 50% on the ventilator support so that the patient may be extubated tomorrow, or can I help [the patient] to wake up a little or do the little extra that is not talked about in the morning but that you know is the right way to go?’ (16).

We found broad agreement in the data that caring for critically ill patients is now tougher for APNs, who thus need *strong mental health*. Due to increased lifespans, the number of people in advanced age needing intensive care has grown, and they stay longer in the unit than the younger population. Furthermore, medical technical equipment prolongs patients' lives, with the consequence that some patients do not die naturally but as a result of end‐of‐life care decisions'. The participants felt great emotional challenges related to caring for critically ill patients with highly complex treatments who, for example, were exposed to trauma or endured long hospital stays (e.g., 3–4 months). The treatment approach has also changed in recent years, so critically ill patients are less sedated than formerly, and the participants reported that these patients endured suffering and anxiety. Consequently, APNs may have to use physical restraint when dealing with agitated and psychotic patients. The participants noted that they used alarms to get help from peers when caring for patients they perceived as threatening.

*Upholding of ethical standards* was highlighted in several statements, as APNs must be fellows and role models for colleagues, be emphatic, treat every patient well and meet patients' and relatives' needs for information. They must also preserve patients' integrity. As one participant said, ‘We are very close to the patient in care. We pick in their face and their mouth, we fix their tube tape, we look into their eyes, we fold up [the duvet], we take away [things], we listen [with stethoscopes], we touch and squeeze with our hands, we use our senses. I feel like we do it all the time, and the patient does not have the opportunity to say, “Now I think you are too close to me,” “Now I want you to cover me,” [or] “I do not want to show this to”—whether it is a wound or …’ (7).

APNs deal daily with ethical conflicts and dilemmas, as many critically ill patients who were not treated 5 years ago are now being treated, and APNs must have the courage to discuss cases and recognise boundaries. Elderly patients who are ‘treated and treated’ are not allowed to say that they want to die, and the following story shows how such an ethical challenge was assessed: ‘I mentioned that I thought we should involve an ethics committee, so I called. The attending physician thought it was unnecessary, because it became so complicated. But I said that this is so difficult, we have a problem. I took it in stride. But when the person [from the ethics committee] came, it was very good. We should do it more often, get it highlighted, because we are nurses, and they are doctors and have their agenda; we have different points of view’ (9).

### 3.2 Advanced clinical decision‐making skills

Under this theme, the subtheme *integration of theory and practice* describes the importance of APNs independently performing examinations by quickly overviewing and identifying the problems of critically ill patients. The APN must seek systematic information by maintaining an airway, breathing, circulation, disability, exposure (ABCDE) assessment mindset when observing and monitoring patients. The participants stressed that an APN should always be prepared, alert and able to identify changes in a critically ill patient's signs and symptoms. Moreover, APNs must possess theoretical knowledge of anatomy, physiology, surgery and mental health. One participant illustrated: ‘There is an interaction between theory and practice, and it is required that, when something occurs, you understand what is happening. It is typical that our day at work is unpredictable. You have a plan, [but] suddenly you must turn everything upside down because there can be acute events like a cardiac arrest. We must always be a little ahead, which requires that you have the knowledge ingrained’ (16). Drawing upon theoretical knowledge and their own experiences, APNs need to perform independent clinical assessments of patients regardless of what is reported during the shift change. ‘You identify the patients’ condition by examining the patients yourself, seeing and touching them. Technical equipment may misinform; observe the lips—does it look like they have 65% saturation, or does it look more like they have 93%?’ (4). APNs may be alone during observations, and it is important that they discuss their assessments with the right professionals at the right time.

The participants stressed that APNs must be able to understand the complexity of a patient's situation and assess whether the care is correctly performed and the desired results obtained. One participant gave an example: ‘If there is an oxygenation problem, it is natural to use Optiflow, and, if there is a “chronic obstructive pulmonal disease”, then the problem is poor ventilation, and the patient needs treatment with non‐invasive ventilation’ (8).

The participants perceived that APNs must be able to deal with *complex combinations of practical and technical skills*. They distinguished between basic and complex skills, with the former including being able to check a bag or suction as a newly credentialed APN and the latter including mastering the organisation of everything happening around the patient. There are many requirements to handle on a shift, and APNs must work on several tasks at once while being prepared for what may happen. They must also handle the situation correctly while communicating clearly with everyone. One participant stated, ‘There is a requirement that we must be able to take care of relatives, take care of children as relatives and be able to communicate with them while the injection needs to be given in this minute, not in the next minute’ (2). Therefore, practising in the critical care unit was perceived as a difficult balancing act. The technical skills may include handling ventilator treatment in a way that makes the patient comfortable, which requires two to three years of clinical work experience in the critical care unit. Current ventilator treatments are perceived as an exciting field in rapid development. According to one of the participants, ‘The ventilators are more advanced, and new functions require more knowledge about lung physiology, ventilator weaning and the effect of changes. Earlier, the ventilators only blew air into the patient; there was no distilling flow; you only had sedation. Now, you handle and think comfort, and the critical care patients are awake; these developments are interesting’ (16).

The work tasks of today's APN involve more preparation of advanced medical technical equipment, such as dialysis machines. One participant explained, ‘It is much tougher now than earlier. Today, you are obliged to deal with an ECMO [extracorporeal membrane oxygenation] machine without backup. Backup is withdrawn because it is saving [money]. In other units, there is less medical technical equipment and therefore more stable conditions and more thorough logistics’ (16).

Regarding *increased delegated responsibility*, several participants believed that their responsibility had increased compared to the past. Responsibility was not static but developed with the addition of new tasks that had previously been performed by physicians or other professionals. For example, nurses working in intensive units have been given more responsibility for oxygen equipment and can use Optiflow without a prescription from a physician. One nurse said, ‘Previously, we only regulated the oxygen supply and did not turn on the ventilator settings without talking to the physician who was responsible for the patient. Today, we are more in dialogue; we change the setting within given limits, give feedback on the effect in order to, for example, change the mode or setting, limiting the ventilator support’ (7). There was a perception among the participants that, provided one likes the challenge and feels safe, the increased responsibility is acceptable.

The data show that APNs must take responsibility within given limits and know what they are willing to do according to the physicians' instructions. An APN may make an independent decision to change the pressure on a respirator if the patient is in the weaning phase, but they must also be able to set limits on their professional responsibilities. One of the participants said, ‘We can suddenly get an order from the surgeon to withdraw an intracranial pressure meter from the patient or take samples from an external ventricular drainage, which are manoeuvres we should not perform, and we do not want to do it, since this equipment goes straight into the brain. If I had recently graduated, it would be difficult to take on that fight [with the physician]’ (12).

APNs must *take the lead in managing increased practice complexity*, including handling and coordinating advanced work tasks. Because critically ill patients are awake when receiving ventilator treatment, they express much fear, anxiety, restlessness, confusion and panic. In addition, conscious patients on ventilator treatment may receive dialysis or an aortic balloon pump treatment and must be helped to sit up and move their legs. This care of critically ill patients requires planning and fortitude as well as the teamwork of many health professionals. One nurse concluded, ‘The APN's role in patient care is a difficult task, leading the patient‐centred work and communicating observations to the physician to initiate a dialogue. We must be very clear and structured and present a well‐founded plan to further promote the treatment. It's overwhelming’ (14). Nurses in critical care did not have to handle such tasks 10 years ago.

### 3.3 Interpersonal skills

*Practising collaboratively* emerged as a very important subtheme in the data; an APN should be able to collaborate with anyone. APNs collaborate with patients' relatives more than before, and, on behalf of the patient, they must convey the relatives' thoughts in interdisciplinary meetings, for example, regarding the patient's basic needs, such as preferred bed rest. APNs must create good dialogue in teams that share common goals with the patient. The participants perceived that they were the initiators of interprofessional meeting requests, but meetings were held infrequently. They often had patients for extended periods with no opportunity to collaborate, which hindered the transfer of knowledge and proposed solutions. On a team, patient treatment strategies may be discussed and disagreements resolved. Good professional argumentation from an APN is required for teamwork, because, in a critical care unit, many health personnel hold strong opinions. As the responsibilities of nurses in critical care have increased, they have a greater opportunity to hold a more equivalent position in dialogue with the responsible physician. Nevertheless, the participants stated that an APN should not make radical changes without consulting a physician, for example, regarding ventilator mode.

Communication and mutual respect were important topics for collaborative practice. All specialisations must collaborate, as everyone wants the best for the patient. One of the participants noted, ‘The APN must take responsibility for creating a caring culture. How you say things is important, and it is essential not to have an arrogant attitude’ (15). The participants agreed on the need to follow the communication structure of identify, situation, background, assessment and recommendation (ISBAR), which gives physicians correct information and the opportunity to prioritise. Using ISBAR also allows APNs to be understood and taken seriously.

In the data, the *ability to position oneself* was related to courage. One participant explained, ‘Courage is based on knowledge and experience; the more I can, the more I venture to share my opinions’ (3). The APN must have the professional influence and courage to argue regarding how the team should work for the patient, but, as one participant said, ‘To voice your opinions and assessments, it is of importance to feel valuable, and you must trust yourself and your own judgements’ (1). The participants had experienced that physicians consequently turned to the most senior nurses in meetings. The participants became animated as they described how physicians exerted their power by not listening in critical situations and instead ordering the nurse to close the door. Nevertheless, the participants expected that APNs must identify, understand and communicate their assessments to the physician should a patient's progress go awry. ‘The whole complexity in critical care units has changed. For APNs, other requirements are set, and critical care is a much larger task than it was before. The physicians’ come in and observe for 5 min and go out again, giving them a completely different perception of the situation. You must have the courage to say that this is not the way we can work, and that is tough, even for us who have been in such situations many times. We support each other and need to take care of the new ones in such situations' (13). This courage is also important in interdisciplinary meetings. For example, when arguing for withdrawal of life‐prolonging treatment, it is important to find questions that may trigger a thought process in the entire treating team, and it is necessary to see the situation from different viewpoints. In difficult treatment situations, the participants recommended contacting the regional ethics committee to establish interprofessional decision‐making processes.

Regarding the subtheme of *peer guidance*, most participants believed that they guided both colleagues and students continually and on several levels, agreeing that ‘if I have something I wonder about, I go to a colleague and ask if we can reflect on the assessment. [I say,] “That's how I think now”, then she can say, “Well, maybe not” or “Yes, I think that sounds reasonable”. Almost everyone does the practical part, thinking aloud. On the more emotional part, if I see someone who is suddenly very emotional, then I think it's part of my job to say that “I see you now; do you want to talk now? Or we could talk later” (13).

Several participants said that they learned a lot from students, and, as preceptors, they had an important role in helping students reflect on the knowledge they need when assessing a given situation. The participants guided students to combine and handle all challenges arising at the same time, from high‐tech treatment to dealing with relatives who are children. As an executive APN, one must intensively evaluate students who may not be suitable as APNs due to anxiety or an inability to feel safe and calm. Preceptors must meet nurse students where they are; some need extensive guidance, others need less and communication is crucial.

## 4 DISCUSSION

This study explored the required competencies of APNs working with increased patients' complexity and comorbidity in critical care units in Norway. Our findings show that APNs must have high‐level competencies including *intrapersonal skills, advanced clinical decision‐making skills* and *interpersonal skills*.

### 4.1 Intrapersonal skills

In the data, we found that APNs must acknowledge their own professional limitations and take personal responsibility for their actions. Insight into one's own competence is important for patient safety and the practice of one's work (Fetro et al., 2010). In the literature, intrapersonal skills include nurses' ability to understand, deal with emotions and practice self‐discipline, which requires motivation and hard work (Fetro et al., 2010). In our data, there was strong agreement among the participants that APNs must be motivated and committed. Motivation is a key to achieve necessary competence from the day the APN students undertake postgraduate studies in critical care, and most often is the motivation for students related to the acquisition of new knowledge and technical skills (Oldland et al., 2023). The motivation to have sufficient technological understanding and competence to manage monitoring and clinical information systems is necessary for APNs daily work (Alenazy et al., 2023). However, the motivation among the APNs in critical care can be affected by their job satisfaction and supportive practice environment (Alenazy et al., 2023). Therefore, the critical care units' leaders need to be aware of supporting their employees and facilitate the development of good relationships to continue developing their intrapersonal skills and upholding motivation, which perform hard work.

In our findings, the APNs' need for strong mental health reflects the great emotional challenges they are exposed to when caring for critically ill, agitated and aggressive patients with high levels of complexity. According to the World Health Organization (2018), experiencing violence may cause risks to mental health, which is in line with our findings. APNs are also regularly exposed to ethical dilemmas, which may lead to the development of burnout, anxiety and depression (Havaei et al., 2021). Thus, strong mental health is essential for nurses to cope with their stress and to work productively. A recent study shows that a great number of CCNs are in a poor state of mental health, highlighting the importance of APNs receiving mental health support from healthcare institutions and their staff managers (Greenberg et al., 2021). When developing mental health programme support, it is important to bear in mind that personality factors also make individuals vulnerable to mental health problems (World Health Organization, 2018). To some degree, the COVID‐19 pandemic showed the world the importance of professional skills in saving lives, yet they remain insufficiently acknowledged. APNs have reported Covid‐19–related staff and equipment shortages across primary and secondary care, and shortages of personal protective equipment during a pandemic are a known factor in the development of mental health sequelae as well as a risk factor for increased turnover and retention problems (Wood et al., 2021). It reflects a systemic failure that some patients in critical care may not receive the necessary elements of evidence‐based care, and there is a risk that patients' needs will not be met (Bourgault, 2022). These problems must be addressed to retain APNs in work life and recruit new nurses to pursue graduate education to acquire a high level of competence.

### 4.2 Advanced clinical decision‐making skills

This study emphasises that APNs in critical care need high‐level competence. These findings align with the description of professional standards established by the International Council of Nurses (2020). A previous study found that APNs' competence embraces several skills, including advocacy, management, leadership, collaboration, communication, consultation, education, ethics, evidence‐based practice, expert clinical judgement, professional autonomy, quality management and research (Sastre‐Fullana et al., 2014). One might argue that these skills are expected from nurse professionals generally, independently of their area of expertise, and not only from APNs. Our study makes the difference explicit by showing that APNs require high‐level competence, so the needed skills are expected to be at a higher level than ordinary registered nurses. In line with Ääri et al. (2008) and Henriksen et al. (2021), we found that APNs must have the capacity and ability to perform their duties in critical care while expressing empathy to patients and their relatives. They must take more responsibility within given limits and take the lead in managing increased practice complexity.

In the data, we found that the participants have assumed more responsibility (transferred from physicians) and have less time to focus on patient well‐being. Recently, educational programmes have evolved slightly to include what an APN in critical care should be able to do after completing a master's programme. APNs in critical care should not act as physicians but cooperate with them. It is crucial, then, that APNs possess the courage and willingness to take a place appropriate to their skills in the team to provide the expected contributions.

### 4.3 Interpersonal skills

This study found that an APN must practice collaboratively with anyone and take responsibility for creating a caring culture; in other words, APNs need interpersonal skills, which involve developing and maintaining relationships through communication, cooperation, empathy and negotiation (Fetro et al., 2010). It is also expected that APNs, offer peer guidance and supervise and evaluate master students, all within a diverse spectrum of contexts as noted by Dunn et al. (2000). Because APNs in critical care must not only care for critically ill patients but also supervise students, they must manage critically ill patients with increased complexity at an advanced level while simultaneously functioning as preceptors. This role needs to be recognised and supported due to its importance for nurturing future APNs and ensuring that they develop the necessary high competence in peer guidance.

APNs' preceptorship role has become even more important because the world is facing a shortage of nurse professionals and a deficit of appropriate formal staff competence (Bourgault, 2022).

### 4.4 Last considerations

Our informants did not talk at length about research, leadership or patient flow and coordination, which are important roles of an APN (Egerod et al., 2021; Tracy et al., 2023). The APN is still a new educational offering in European countries (Egerod et al., 2021), and, as the APN role has only recently been established in the Norwegian education programme, it will take some time before the findings regarding APNs' needed skills are perceived in clinical practice. Today's APNs are educated in evidence‐based practice but often have no time for development projects or research in their daily work. It may be assumed that APN's research skills will be used more in clinical practice as their role is clarified.

### 4.5 Strengths and limitations

A strength of this study is that the focus group interviews were conducted mainly with highly experienced CCNs in four different critical care units. In total, 14 of the 18 participants were experts in their wards, and the other four participants were educated as APNs in critical care (Table 3). In addition, this study was developed, completed, analysed and written in collaboration with clinicians and educators who are all APNs in critical care. One limitation of the study is that the findings represent only four critical care units in Norway, but these units are characterised by the highest degree of medical complexity in Norway. It may also be a limitation that we did not interview the nursing managers and the physicians, who are a part of the daily teamwork in the wards. Nursing managers might offer executive perspectives on what APNs require in terms of competence, and physicians might provide supervision and oversight of APN practice care.

## 5 CONCLUSION

APN students in critical care develop their needed professional competence in extremely complex and interactive situations. In the care of vulnerable acute and critically ill patients, APNs in critical care must possess high‐professional competence that allows them to simultaneously manage multiple complex and challenging work tasks. As managing complex patient situations includes the growing problem of agitated patients, we suggest that educational institutions should give more attention to APN students to develop intrapersonal skills, including self‐awareness, motivation and strong mental health. Further interpersonal‐ and advanced clinical decision‐making skills are important to develop and ensure the acquisition of the required competence level. Further studies should involve the physicians view of the APNs professional competence.

## AUTHOR CONTRIBUTIONS

Four of the authors (MTS, CM, IJF and PKL) chose the design and method and agreed on a progress plan. MTS planned and conducted the focus group interviews and instructed the co‐moderators (CM and IJF). MTS moderated the four focus group interviews. MTS and CM subsequently transcribed verbatim three and one interviews, respectively. The analysis was performed by MTS, CM, IJF and PKL, with valuable contributions from IP and AAGN. MTS wrote the article, and all the authors (MTS, IP, AAGN, CM, IJF and PKL) contributed to critically revising the manuscript for content and have given final approval to publish in the *Nursing Open*.

## FUNDING INFORMATION

This research did not receive any specific grant from funding agencies in the public, commercial or non‐profit sectors.

## CONFLICT OF INTEREST STATEMENT

The authors declare no conflicts of interest.

## Data Availability

The data that support the findings of this study are available from the corresponding author upon reasonable request.
